# B-Myb Is Up-Regulated and Promotes Cell Growth and Motility in Non-Small Cell Lung Cancer

**DOI:** 10.3390/ijms18060860

**Published:** 2017-05-27

**Authors:** Yuelei Jin, Huifang Zhu, Wei Cai, Xiaoyan Fan, Yitao Wang, Yulong Niu, Fangzhou Song, Youquan Bu

**Affiliations:** 1 Department of Biochemistry and Molecular Biology, Chongqing Medical University, 1# Yixueyuan Road, Yuzhong District, Chongqing 400016, China; jinyuelei_001@163.com (Y.J.); zhu_huifang@126.com (H.Z.); caiwei5300@163.com (W.C.); fanxiaoyan1115@163.com (X.F.); wytao8899@126.com (Y.W.); 2 Molecular Medicine and Cancer Research Center, Chongqing Medical University, Chongqing 400016, China; maggie8818@sina.com

**Keywords:** B-Myb, lung cancer, proliferation, migration, extracellular signal-regulated kinases (ERK)

## Abstract

B-Myb is a transcription factor that is overexpressed and plays an oncogenic role in several types of human cancers. However, its potential implication in lung cancer remains elusive. In the present study, we have for the first time investigated the expression profile of B-Myb and its functional impact in lung cancer. Expression analysis by quantificational real-time polymerase chain reaction (qRT-PCR) and immunohistochemistry demonstrated that B-Myb expression is aberrantly overexpressed in non-small cell lung cancer (NSCLC), and positively correlated with pathologic grade and clinical stage of NSCLC. A gain-of-function study revealed that overexpression of B-Myb significantly increases lung cancer cell growth, colony formation, migration, and invasion. Conversely, a loss-of-function study showed that knockdown of B-Myb decreases cell growth, migration, and invasion. B-Myb overexpression also promoted tumor growth in vivo in a NSCLC xenograft nude mouse model. A molecular mechanistic study by RNA-sequencing (RNA-seq) analysis showed that B-Myb overexpression causes up-regulation of various downstream genes (e.g., *COL11A1*, *COL6A1*, *FN1*, *MMP2*, *NID1*, *FLT4*, *INSR*, and *CCNA1*) and activation of multiple critical pathways (e.g., extracellular signal-regulated kinases (ERK) and phosphorylated-protein kinase B (Akt) signaling pathways) involved in cell proliferation, tumorigenesis, and metastasis. Collectively, our results indicate a tumor-promoting role for B-Myb in NSCLC and thus imply its potential as a target for the diagnosis and/or treatment of NSCLC.

## 1. Introduction

Lung cancer is the most common incident cancer and remains the leading cause of cancer-related deaths in China and worldwide [1,2,3]. The two major forms of lung cancer are non-small cell lung cancer (NSCLC, about 85% of all lung cancers) and small-cell lung cancer (SCLC, about 15%). NSCLC is further classified into three major histologic subtypes: squamous-cell carcinoma (SQCC), adenocarcinoma (ADC), and large-cell lung cancer. Although significant advances have been achieved during the past few decades, the prognosis for patients with lung cancer remains relatively poor, with an overall five-year survival rate of about 18% [2]. Thus, to develop novel approaches and strategies for the diagnosis and treatment of this lethal disease, more efforts are needed to investigate the complicated molecular mechanisms underlying the initiation and progression of this malignancy. 

B-Myb, also known as MYB proto-oncogene like 2 (MYBl2), is a transcription factor that belongs to the Myb gene family including A-Myb and c-Myb [4,5,6,7,8,9,10,11,12]. While A-Myb and c-Myb are tissue-specific, B-Myb is widely expressed in rapidly dividing cells [6], and is crucially involved in cell proliferation [13], control of cellular differentiation [14], regulation of apoptosis [15], and tumor progression [16]. B-Myb stimulates transcription of genes that promote entry into the S- and M-phases of cell cycle [17]. B-Myb has been shown to be overexpressed in a broad range of human cancers, such as breast cancer [18], hepatocellular carcinoma [10], lung cancer [19], colon cancer [20], neuroblastoma [21], and T-cell lymphomas [22]. Moreover, B-Myb has prognostic value for predicting outcomes in patients with several types of cancers, including breast cancer and acute myeloid leukemia [23]. To date, the expression profile of B-Myb and its potential functional impact in lung cancer have not been investigated and remain elusive. 

In the present study, we have for the first time found that B-Myb expression was significantly up-regulated in NSCLC, and closely correlated with clinicopathologic features of NSCLC. A gain-of-function study demonstrated that overexpression of B-Myb significantly increased lung cancer cell growth and motility, whereas a loss-of-function study revealed opposite phenotype changes. A molecular mechanistic study showed that B-Myb overexpression could enhance the activation of extracellular signal-regulated kinases (ERK) and phosphorylated-protein kinase B (Akt) signaling pathways. Thus, our findings reveal a potential tumor-promoting role of B-Myb in NSCLC. 

## 2. Results

### 2.1. B-Myb Expression Is Up-Regulated in NSCLC

**Figure 1:**
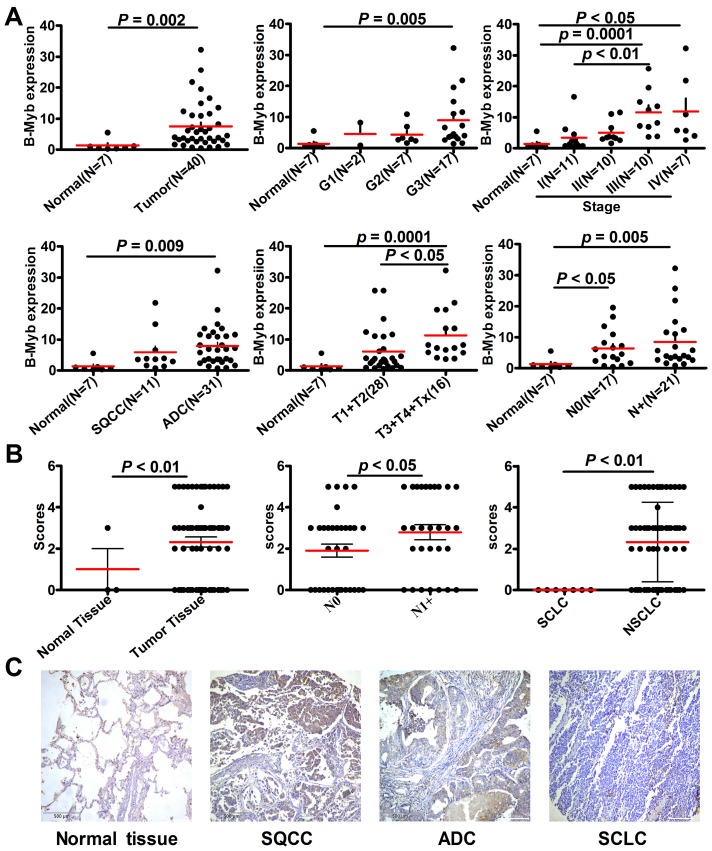
B-Myb expression is up-regulated in NSCLC. (**A**) Overexpression of B-Myb mRNA in lung cancer. Expression of B-Myb was determined by quantificational real-time polymerase chain reaction (qRT-PCR) with specific primers in Origene TissueScan Lung Cancer Panels (HLRT103). Grades: G1, G2, and G3. Stages: I, II, III, and IV. N0, non-lymph node metastatic; N+, lymph node metastatic. (**B**) Overexpression of B-Myb protein in lung cancer. Expression of B-Myb protein was examined by immunohistochemical analysis using lung cancer tissue microarray slides. (**C**) Representative immunohistochemical images of B-Myb expression in lung cancer (scale bar: 500 μm). SQCC, lung squamous cell carcinoma; ADC, lung adenocarcinoma; SCLC, small cell lung cancer; NSCLC, non-small cell lung cancer.

In the first step of this study, we determined the expression profile of B-Myb in lung cancer. The quantificational real-time polymerase chain reaction (qRT-PCR) analysis on a panel of lung cancer complementary DNA (cDNA) arrays including 40 patients with lung cancer and seven healthy controls demonstrated that B-Myb was significantly up-regulated at the mRNA level in lung cancer samples compared with normal lung tissues (*p* = 0.002; Figure 1A). Analysis of B-Myb expression in the context of various clinicopathologic features revealed that the expression level of B-Myb mRNA was positively correlated with pathologic grade (*p* = 0.005), clinical stage (*p* < 0.05), and tumor-node-metastasis (TNM) classification (*p* < 0.01; Figure 1A). B-Myb mRNA was remarkably upregulated in two of the main NSCLC subtypes (i.e., SQCC and ADC (*p* = 0.009; Figure 1A)). Notably, B-Myb mRNA levels were significantly higher in samples with metastasis compared with primary tumors (*p* = 0.015; Figure S1). 

Consistently, immunohistochemistry analysis on a lung cancer tissue microarray showed that B-Myb was also significantly up-regulated at the protein level in NSCLC samples compared with normal lung tissues (*p* < 0.01; Figure 1B,C; Table 1). Notably, expression of B-Myb protein was undetectable in all seven SCLC tissues (Figure 1C; Table 1). B-Myb protein levels were higher in NCSLC tissues with lymph node metastasis (pN0) compared with that without lymph node metastasis (pN1+) (*p* < 0.05; Figure 1B; Table 1). Taken together, these results clearly show that B-Myb is up-regulated in NCSLC, and hence points to a potential tumor-promoting function for B-Myb in NCSLC. 

### 2.2. B-Myb Increases Lung Cancer Cell Growth

**Figure 2:**
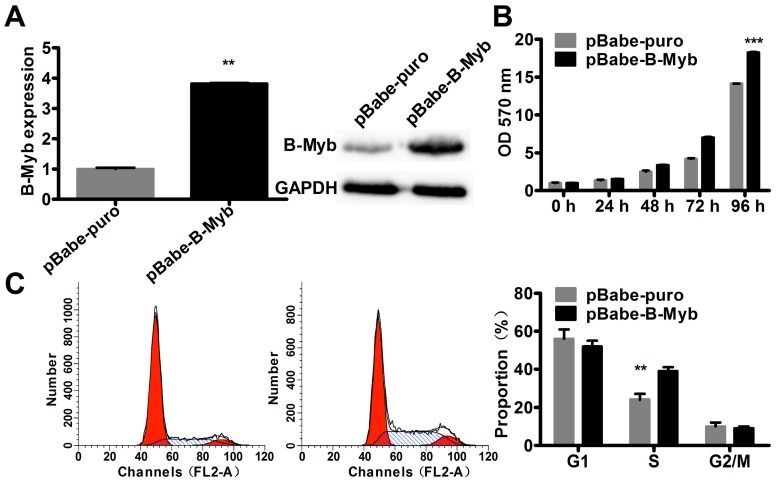
Overexpression of B-Myb promotes lung cancer cell proliferation and cell cycle progression. (**A**) Stable overexpression of B-Myb. H1299 lung cancer cells were transfected with pBabe.puro.GWrfA empty vector and pBabe.puro.GWrfA-B-Myb expression vector. Cells were then selected with puromycin to establish the monoclonal stable control (pBabe-puro) and B-Myb overexpression (pBabe-B-Myb) cell lines. Expression of B-Myb was determined by qRT-PCR and immunoblot analysis. (**B**) B-Myb overexpression stimulates cell proliferation. Cell proliferation was monitored by methylthiazol tetrazolium (MTT) assay in the stable control and B-Myb overexpression cells at the indicated time-points. (**C**) B-Myb overexpression promotes S-phase progression. The stable control and B-Myb overexpression cells were seeded on six-well plates, and twenty-four hours later cells were collected and subjected to cell cycle distribution analysis. The data are presented as the mean and standard deviation (SD) of triplicates from a representative experiment. ** *p* < 0.01, *** *p* < 0.001.

We then sought to determine whether B-Myb could increase lung cancer cell growth. For this purpose, H1299 lung cancer cells were transfected with pBabe-B-Myb expression vector or the pBabe control vector to establish the corresponding stable cell lines, pBabe-B-Myb and pBabe-control. As shown in Figure 2A, B-Myb expression was significantly increased at both mRNA and protein levels in pBabe-B-Myb overexpression stable cells compared with pBabe-control stable cells. Cell proliferation assay revealed that overexpression of B-Myb significantly promoted cell growth (Figure 2B). Cell cycle analysis demonstrated that B-Myb overexpression caused a significant increase in the percentage of S-phase cells compared with the control cells showing low B-Myb expression (Figure 2C). 

**Figure 3:**
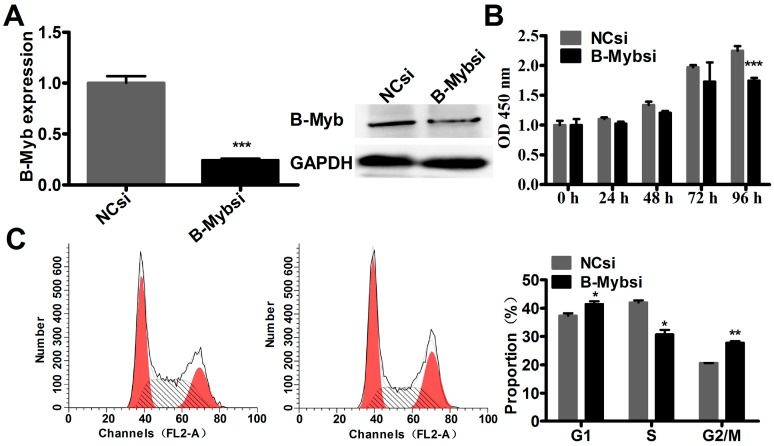
Knockdown of B-Myb inhibits lung cancer cell proliferation and cell cycle progression. (**A**) small interfering RNA (siRNA)-mediated knockdown of B-Myb. H1299 cells were transfected with negative control siRNA (NCsi) and siRNA against B-Myb (B-Mybsi). Twenty-four hours after transfection, cells were collected, and total RNA and whole cell lysates were prepared. Expression of B-Myb was determined by qRT-PCR and immunoblot analysis. (**B**) B-Myb knockdown inhibits cell proliferation. H1299 cells were transiently transfected with negative control or B-Myb siRNA, and cell proliferation was then monitored by Cell Counting Kit-8 assay kits (CCK8) at the indicated time-points. (**C**) B-Myb knockdown delays S-phase progression. H1299 cells were seeded on six-well plates, transfected with the indicated siRNAs, and twenty-four hours later cells were collected and subjected to cell cycle analysis. Data represent the mean ± SD. All experiments were performed in triplicates. * *p* < 0.05, ** *p* < 0.01, *** *p* < 0.001.

To further confirm the above results obtained by the gain-of-function study, a loss-of-function study was conducted using specific small interfering RNA (siRNA) to knockdown the endogenous expression of B-Myb in H1299 cells. As shown in Figure 3, endogenous expression of B-Myb was remarkably silenced at both mRNA and protein levels in cells transfected with B-Myb siRNA compared with that with negative control siRNA. Cell proliferation assay and cell cycle analysis revealed that knockdown of B-Myb decreased cell growth, and caused a significant decrease in the percentage of S-phase cells accompanied by a G2/M arrest (Figure 3). Taken together, these gain-of-function and loss-of-function results clearly indicate that B-Myb increases lung cancer cell proliferation at least partially through accelerating S-phase progression. 

**Figure 4:**
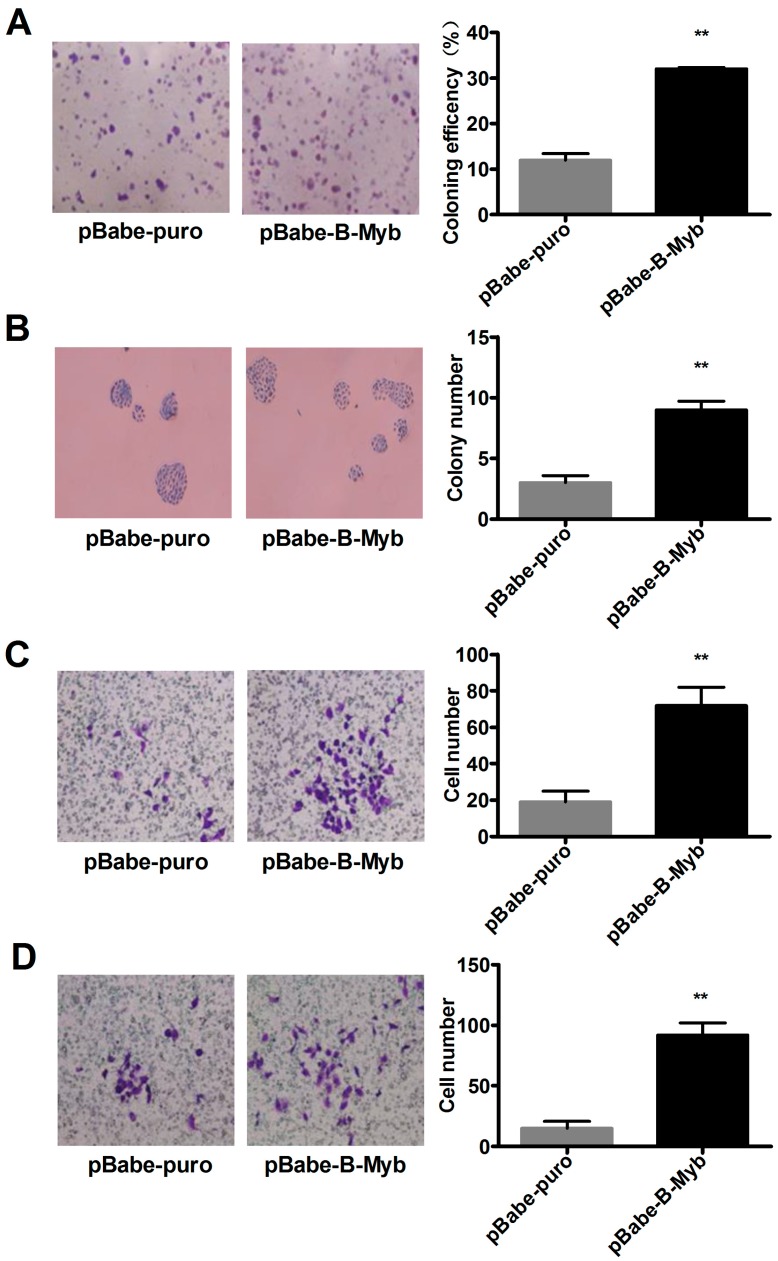
B-Myb overexpression increases lung cancer cell colony formation, migration, and invasion. (**A**,**B**) B-Myb enhances colony formation. The stable control (pBabe-puro) and B-Myb overexpression (pBabe-B-Myb) H1299 cells were seeded on plastic plates for anchorage-dependent colony formation assay (**A**) and soft agar for anchorage-independent colony formation assay (**B**), respectively. (**C**) B-Myb overexpression promotes cell migration. A Transwell migration assay was conducted with the pBabe-puro and pBabe-B-Myb stable cells in the Transwell devices without pre-coating Matrigel, as described in detail in the Materials and Methods section. Representative images (×200) (left) and quantification results (right) were shown. (**D**) B-Myb overexpression promotes cell invasion. A Transwell invasion assay was conducted with the indicated cells in the Transwell devices with pre-coating Matrigel, as described in detail in the Materials and Methods section. Representative images (×200) (left) and quantification results (right) were shown. ** *p* < 0.01.

In addition, in support with the results obtained from the cell growth assay, colony formation assays on plastic and soft agar further demonstrated that overexpression of B-Myb in H1299 cells also remarkably enhanced anchorage-dependent and -independent colony forming ability compared with the control cells (Figure 4A,B). These data suggest that B-Myb promotes lung tumorigenesis in vitro. 

### 2.3. B-Myb Promotes Lung Cancer Cell Migration and Invasion

**Figure 5:**
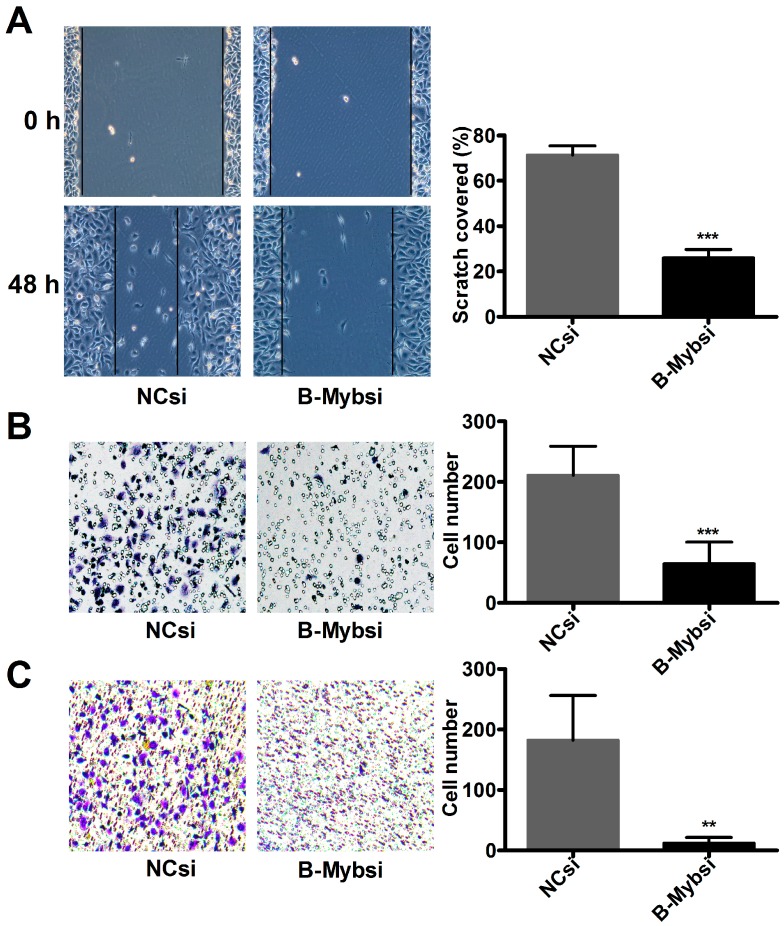
B-Myb knockdown decreases cell migration and invasion. B-Myb knockdown inhibits cell migration. H1299 cells were transiently transfected with negative control siRNA and siRNA against B-Myb, and then subjected to (**A**) a wound healing assay, (**B**) a Transwell migration assay, and (**C**) a Transwell invasion assay, as described in the Materials and Methods section, respectively. ** *p* < 0.01, *** *p* < 0.001.

Next, we analyzed the effect of B-Myb overexpression on lung cancer cell migration and invasion. As shown in Figure 4C, a Transwell migration assay demonstrated that overexpression of B-Myb in H1299 cells caused a significant increase in the number of cells that penetrated the Transwell chamber membrane compared with the control cells. Furthermore, a Transwell invasion assay revealed that B-Myb overexpression remarkably increased the number of cells that invaded through the Matrigel-coated membrane (Figure 4D). Conversely, knockdown experiments revealed that siRNA-mediated silencing of B-Myb significantly inhibited the cell migration and invasion abilities, as evidenced by the wound healing assay, and Transwell migration and invasion assays (Figure 5A–C). Collectively, these results strongly suggest that B-Myb promotes both migration and invasion abilities of lung cancer cells. 

### 2.4. B-Myb Enhances Lung Tumorigenesis In Vivo

**Figure 6:**
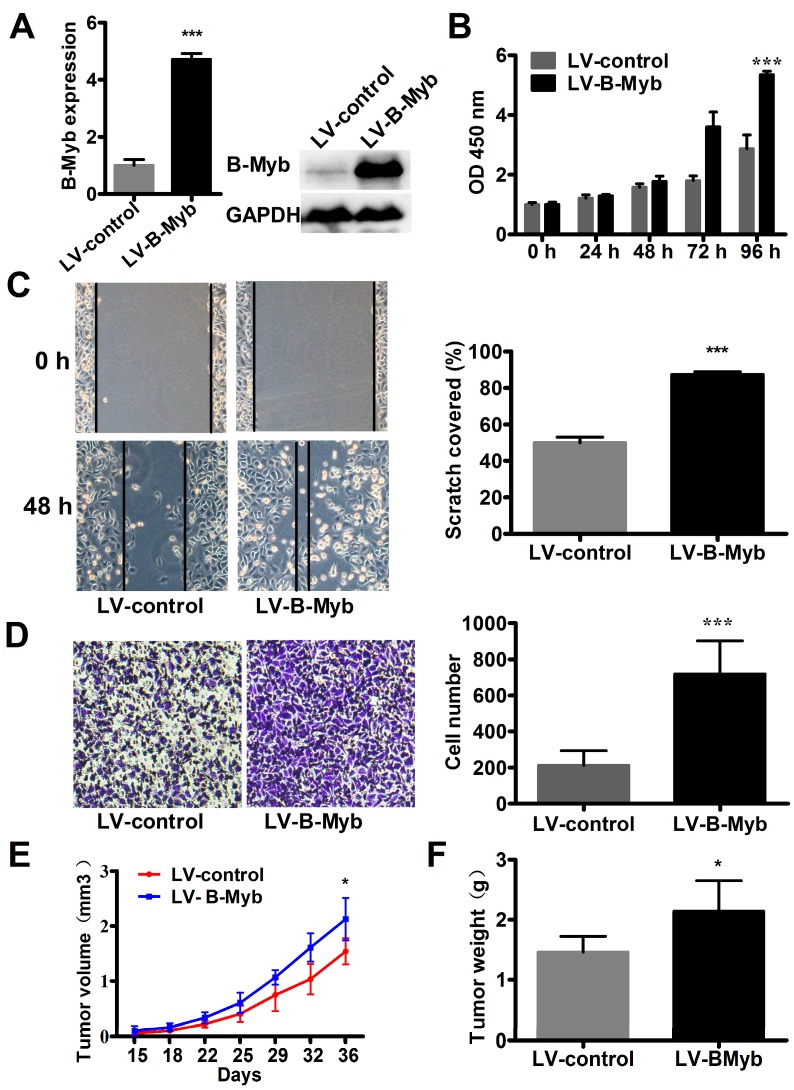
B-Myb overexpression promotes lung tumorigenesis in vitro and in vivo. (**A**) Lentivirus-mediated B-Myb overexpression. H1299 cells were infected with the control lentiviral particles and that led to expressing B-Myb, and then selected in the presence of puromycin to obtain the polyclonal control (LV-control) and B-Myb overexpression (LV-B-Myb) stable cells. Expression of B-Myb was examined by qRT-PCR and immunoblot analysis. (**B**) B-Myb increases cell proliferation. Cell proliferation was monitored by CCK8 assay in the stable control and B-Myb overexpression cells at the indicated time-points. (**C**) and (**D**) B-Myb augments cell motility. The stable control and B-Myb overexpression cells were seeded on six-well plates for the wound healing assay (**C**) and Transwell chamber for the Transwell migration assay (**D**), respectively. Representative images (×200) (left) and quantification results (right) were shown for each assay. (**E**) and (**F**) B-Myb promotes lung tumor growth in vivo. The polyclonal control (LV-control) and B-Myb overexpression (LV-B-Myb) stable cells were injected subcutaneously into the dorsal flanks of five nude mice, respectively. The tumor size was measured about twice a week for tumor growth curve construction (**E**). The tumor weight was measured at the end of the experiment (**F**). Data represent the mean ± SD. All experiments were performed in triplicates. * *p* < 0.05, *** *p* < 0.001.

As the above gain-of-function results were mainly obtained from a single stable colony overexpressing B-Myb, the phenotypic changes could be artificial and unreliable. Therefore, to further confirm the results obtained by pBabe vector-mediated B-Myb stable overexpression, we alternatively prepared lentiviral particles expressing B-Myb to establish stable polyclonal cells overexpressing B-Myb (LV-B-Myb). As shown in Figure 6A, B-Myb expression was indeed significantly up-regulated in LV-B-Myb overexpression stable cells compared with LV-control stable cells. Consistent with the results observed in pBabe-mediated monoclonal overexpression system, overexpression of B-Myb also significantly increased cell growth and motility in the lentivirus-mediated polyclonal overexpression system, as evidenced by proliferation, would healing, and Transwell migration assays (Figure 6B–D). 

To further determine whether B-Myb promotes lung tumorigenicity in vivo, the stable LV-B-Myb overexpression cells as well as LV-control cells were subcutaneously injected into the dorsal flank of nude mice, respectively. Tumor growth was then monitored over five weeks. The results showed that both tumor volume (Figure 6E) and tumor weight (Figure 6F) were significantly enhanced in the nude mice with stable B-Myb overexpression, suggesting that B-Myb enhances lung tumor growth in vivo. 

### 2.5. B-Myb Regulates Various Downstream Genes and Pathways

**Figure 7:**
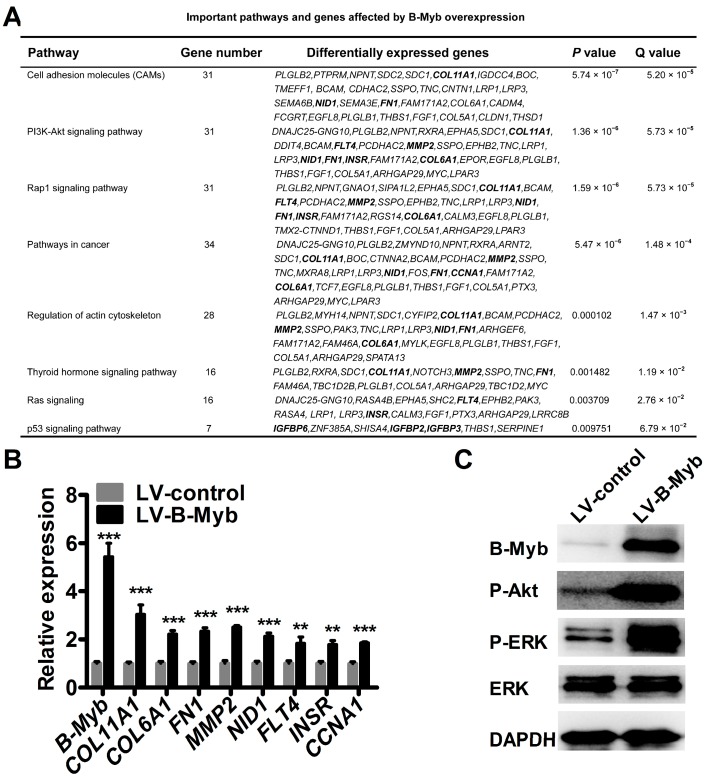
B-Myb overexpression affects various downstream genes and important pathways. (**A**) Important pathways and genes affected by B-Myb overexpression. The stable control (LV-control) and B-Myb overexpression (LV-B-Myb) H1299 cells were subjected to RNA-sequencing (RNA-seq) analysis. Differentially expressed genes between LV-control and LV-B-Myb cells were filtered and subjected to Gene Ontology and pathway analysis, as described in the Materials and Method sections. (**B**) Validation of differentially expressed genes by qRT-PCR. (**C**) B-Myb overexpression activates phosphorylation of ERK and Akt. Exponentially grown LV-control and LV-B-Myb cells were collected and subjected to immunoblot analysis with the indicated antibodies. ** *p* < 0.01, *** *p* < 0.001; GAPDH: glyceraldehyde-3-phosphate dehydrogenase.

To further investigate the potential molecular mechanism underlying B-Myb-mediated tumorigenesis, RNA-sequencing (RNA-seq) analysis was conducted to compare the differential gene expression profiles between stable LV-B-Myb overexpression and LV-control H1299 cells. Differential gene expression analysis revealed that, in total, 390 genes were differentially expressed, including 300 genes that were up-regulated (ratio, >2.0) and 90 genes that were down-regulated (ratio, <0.5) in response to B-Myb overexpression. Gene Ontology analysis revealed that the differentially expressed genes are enriched in extracellular matrix, growth factor binding, developmental process, etc. (Table S1). Pathway analysis on the dysregulated genes affected by B-Myb overexpression identified signaling pathways including cell adhesion molecules, phosphatidyl inositol-3 kinase (PI3K)-Akt signaling pathway, pathways in cancer, and Ras signaling pathway (Figure 7A). The genes in these pathways include, among others, *COL11A1*, *COL6A1*, *FN1*, *MMP2*, *NID1*, *FLT4*, *INSR*, and *CCNA1*, which are known to be involved in proliferation, tumorigenesis, and metastasis. Quantitative RT-PCR analysis further confirmed that these genes were indeed up-regulated in stable LV-B-Myb overexpression cells compared with LV-control cells (Figure 7A). Notably, the qRT-PCR results corresponded well to the RNA-seq data (Figure 7B). Moreover, an immunoblot analysis demonstrated that overexpression of B-Myb significantly increased the levels of phosphorylated ERK and Akt. Taken together, these findings strongly suggest that B-Myb might promote lung cancer development at least partially though positive regulation of ERK and Akt signaling pathways. 

## 3. Discussion

Previous studies have shown that B-Myb is overexpressed in human malignancies and has prognostic value in several different types of cancers, indicating its functional implication in cancer development [4,5,6,7,8]. Ren et al. reported that B-Myb is significantly overexpressed in colorectal cancer tissues compared to adjacent non-cancerous tissues, and B-Myb overexpression is an independent prognostic factor for poor colorectal cancer patient survival, as evidenced by cox multivariate analysis [9]. Nakajima et al. reported firstly that B-Myb is overexpressed in primary hepatocellular carcinoma (HCC) [12]. Calvisi et al. later showed that B-Myb is progressively up-regulated during HCC development and progression, and that the highest expression levels of B-Myb are associated with poorer outcomes in HCC patients [10]. Functional analysis revealed that overexpression of B-Myb increases cell proliferation and cell cycle progression in HCC cells [11]. Our data indicated for the first time that B-Myb expression is significantly up-regulated in NSCLC, and closely correlates with clinicopathologic parameters of NSCLC. A gain-of-function study demonstrated that overexpression of B-Myb significantly induces lung cancer cell growth, migration, and invasion, whereas a loss-of-function study revealed the opposite phenotype changes. Thus, our findings in NSCLC are well consistent with the aforementioned observations obtained from other types of cancers, and thus highly suggest a general tumor-promoting role of B-Myb in cancer development. Of note, the prognostic value of B-Myb expression and therapeutic potential of targeting B-Myb in NSCLC should be further explored, which is a research topic currently under investigation in our lab. 

Previous studies have also shown that B-Myb mechanistically facilitates accelerated growth and progression of malignancies through deregulation of cell cycle and activation of genes and pathways related to tumor progression [4,5,6,7,8,11]. Consistently, our results showed that B-Myb overexpression caused a significant increase in the number of S-phase cells. Moreover, our molecular mechanistic study by RNA-seq analysis revealed that B-Myb overexpression caused up-regulation of various downstream genes such as *CCNA1*, *COL11A1*, *COL6A1*, *MMP2*, *NID1*, and *FLT4*. *CCNA1* is a well-known cyclin that regulates G1/S transition through cyclin A/Cdk2 complex [24,25]. Previous studies have shown that B-Myb transactivates *CCNA1* gene promoter through a Sp1 binding site-dependent mechanism, and *CCNA1* could further directly interact with B-Myb to transactivate B-Myb-regulated genes in some cell lines [24,25]. Thus, it is highly likely that a *B-Myb-CCNA1* regulatory loop also exists and plays an important role in NSCLC cells. *COL11A1*, *COL6A1*, *MMP2*, *NID1*, and *FLT4* have been shown to have a positive role in regulating motility and invasion of various cancer cells [26,27,28,29,30,31]. Moreover, we also found that the deregulated genes affected by B-Myb overexpression could be enriched in ERK and Akt signaling pathways. Of note, previous reports showed that *COL11A1* elevates phosphorylated Akt in ovarian cancer cells by stabilizing *PDK1* [26], and *FLT4* participates in the induction of phosphorylation of extracellular signal-regulated kinase (ERK) 1/2 as well as in the proliferative and migratory ability of SG-2 cells [31]. Our recent data revealed that *NID1* induces ERK phosphorylation in ovarian cancer cells (unpublished to date) [32]. Thus, further studies are needed to investigate whether B-Myb activates ERK and Akt signaling pathways at least partially though up-regulation of *FLT4*, *COL11A1*, and *NID1* in lung cancer cells. 

In summary, our present study demonstrated for the first time that B-Myb is aberrantly overexpressed in NSCLC and that B-Myb promotes lung cancer cell growth both in vitro and in vivo. Our study suggests B-Myb might serve as a potential target in the diagnosis and/or treatment of NSCLC. 

## 4. Materials and Methods

### 4.1. Cell Culture

Human lung cancer cell line H1299 (Chinese Academy of Sciences Shanghai cell bank, Shanghai, China) was grown in a humidified atmosphere containing 5% CO_2_ at 37 °C in Roswell Park Memorial Institute (RPMI)1640 medium supplemented with 50 units/mL penicillin, 50 mg/mL streptomycin, and 10% fetal bovine serum (FBS) (Invitrogen, Carlsbad, CA, USA). 

### 4.2. siRNA Synthesis and Transfection

Small interfering RNA against B-Myb (B-Mybsi) and the negative control siRNA (NCsi) with non-functioning sequences were chemically synthesized by GenePharma (Shanghai, China). The sequences of the B-Myb siRNA are as follows: 5′-CAGACAAUGCUGUGAAGAATT-3′ (sense) and 5′-UUCUUCACAGCAUUGUCUGTT-3′ (antisense). The sequences of NC siRNA are as follows: 5′-UUCUCCGAACGUGUCACGUTT-3′ (sense) and 5′-ACGUGACACGUUCGGAGAATT-3′ (antisense). Lipofectamine RNAiMAX reagent (Invitrogen, Carlsbad, CA, USA) was used for siRNA transient transfection according to the manufacturer’s instructions. Cells were collected and subjected to subsequent analysis 48 h to 72 h after transfection.

### 4.3. RNA Isolation and RT-PCR

Total RNA isolation and quantitative RT-PCR were conducted as described previously [33]. Tissue Scan Lung Cancer quantitative PCR Panels (HLRT103) containing prepared cDNAs were purchased from OriGene (Rockville, MD, USA) and directly used for RT-PCR. RT-PCR was carried out by using the SYBR Premix Ex Taq™ (Perfect Real Time, TAKARA, Otsu, Japan) following the manufacturer’s instructions. The sequences of the primers used can be found in Table S2. 

### 4.4. Tissue Microarrays and Immunohistochemistry

Lung cancer tissue microarray slide (TC0132) was purchased from Auragene Bioscience Corporation (Hunan, China). The tissue microarray contains adenocarcinoma (22 cases), papillary adenocarcinoma (six cases), squamous cell carcinoma (26 cases), small cell carcinoma (seven cases), large cell carcinoma (two cases), atypical carcinoid (six cases), and normal lung tissue (three cases). Detailed information of the tissue microarray (TC0132) is provided in Table S3. Immunohistochemistry was conducted using the PowerVision (PV) two-step method. Briefly, a slide was firstly deparaffinized and rehydrated with xylene and graded alcohol, followed by treatment with 3% H_2_O_2_ for 10 min to block the endogenous peroxidase activity. Then, the slide was boiled with a buffer containing 0.01 mM sodium citrate (pH 6.0) for 15 min for epitope retrieval, followed by pre-incubation with blocking solution (2% BSA (bovine serum albumin)) for 30 min at room temperature. Immunodetection was then performed using the EnVision DAB color kit purchased from Beyotime Biotechnology (Shanghai, China). Information regarding the antibodies used is provided in Table S4. The staining results of tissue microarray slide were categorized as follows: the final staining scores (0–7) were calculated based on the sum of the intensity (0, negative; 1, weak; 2, moderate; and 3, strong) and distribution scores (0, 0%; 1, 1–25%; 2, 26–50%; 3, 51–75%; and 4, 76–100% of cells). Tumors having a final staining score of 0 were negative and those with scores ≥1 were positive. 

### 4.5. pBabe-Mediated Establishment of Stable B-Myb Overexpression Cell Line

pBabe.puro.GWrfA empty vector and pBabe.puro.GWrfA-B-Myb expression vector were kindly offered as gift by Charles M. Pero [18]. H1299 cells were seeded into six-well plates, and transiently transfected with 2 μg of the empty vector or B-Myb expression vector using Lipofectamine 2000 reagent (Invitrogen) according to the manufacturer's instructions. Six hours after transfection, the cells were sub-seeded into 96-well plates at a density of about one cell per well and selected in the presence of 1.3 µg/mL puromycin for generating the control and B-Myb overexpression stable clones. The survived puromycin-resistant cell clones were then continuously expanded and identified to establish the corresponding stable cell lines. 

### 4.6. Lentivirus-Mediated Establishment of Stable B-Myb Overexpression Cell Line

The lentiviral empty vector (EX-NEG-LV105) and human B-Myb expression vector (EX-B0073-LV105-5) were purchased from FulenGen (Guangzhou, China). To produce recombinant lentiviruses, 293Ta cells were co-transfected with the lentiviral empty vector or B-Myb expression vector along with the Lenti-Pac™ HIV Expression Packaging Kit (GeneCopoeia, Guangzhou, China) according to the manufacturer’s directions. Twelve hours after transfection, the culture fluid was replaced by fresh Dulbecco’s modified Eagle’s medium (DMEM) containing 5% of FBS and 0.2%Titer Boost Viral Titer Reagent (500×) (GeneCopoeia) to remove cell debris. Forty-eight hours after transfection, the culture supernatant was collected, concentrated by polyethylene glycol precipitation, filtrated through 0.45 μm cellulose acetate filters, and subjected to centrifugation at 4 °C, 3500 rpm, for 40 min. The obtained lentiviral particles were stored at −80 °C. For lentivirus infection, H1299 cells were seeded on six-well plates at a density of 3 × 10^5^ cells per well. Twenty-four hours after seeding, the culture fluid was replaced by 1900 μL of fresh RPMI 1640 medium containing 5% of FBS together with 100 μL of either control or B-Myb expression lentiviral particles. Stable populations were then selected with culturing in 1.3 μg/mL of puromycin for six days.

### 4.7. Immunoblot Analysis

Immunoblot analysis was performed as described previously [33]. The information regarding the antibodies used in this study is provided in Table S4. The blots were visualized by enhanced chemiluminescence (ECL; Bio-Rad Laboratories, Hercules, CA, USA). 

### 4.8. Cell Proliferation Assay

Cell proliferation was determined by either methylthiazol tetrazolium (MTT) (Beyotime, Shanghai, China) or cell counting kit-8 (CCK8; Dojindo, Tokyo, Japan). Cells were seeded in 96-well plates at a density of 0.2 × 10^4^ cells per well, and 10 μL of MTT solution or CCK8 regent was then added to each well at the indicated time points. The plates were incubated for 4 h at 37 °C, and the absorbance value (OD) of each well was measured at 570 nm for MTT or 450 nm for CCK8 according to the manufacturer’s instructions. The experiments were repeated three times. 

### 4.9. Cell Cycle Analysis

For cell cycle analysis, cells were collected by trypsin digestion and low speed centrifugation, stained using Cell Cycle Kit (Key GEN, Nanjing, China), and then subjected to cell cycle distribution analysis on a FACScan flow cytometer (BD Biosciences, San Jose, CA, USA).

### 4.10. Colony Formation Assay

For anchorage-dependent colony formation assay, H1299 cells were seeded in six-well plates at a density of 2000 cells per well, and continuously cultured for ten days. The resultant cell clones were then fixed with 4% paraformaldehyde for 15 min, stained with 0.5% crystal violet for 10 min, and counted. On the other hand, the anchorage-independent soft agar assay was conducted as described previously with minor modifications [33]. 

### 4.11. Wound Healing Assay

H1299 cells were seeded in six-well plates and cultured. When the cells reached 70% confluence, artificial wounds were firmLy made with a micropipette tip, and floating cells and cell debris were removed by washing cells with phosphate buffered saline (PBS). The cells were then incubated in medium containing 3% fetal bovine serum (Invitrogen). Representative images of cells migrating into the wounds were captured at 0 h, 24 h, and 48 h in the same wounded region under an inverted microscope (Olympus, Hamburg, Germany). 

### 4.12. Transwell Cell Migration and Invasion Assays

For Transwell cell migration and invasion assays, H1299 cells were seeded in the upper chamber of a Transwell device (8 µm, Merck Millipore, Darmstadt, Germany) at a density of 2 × 10^4^ (for migration) or 4 × 10^4^ (for invasion) cells per well in serum-free medium with or without pre-coating Matrigel (BD Biosciences), and 700 μL of medium containing 10% FBS was added to the bottom chamber. Thirty-six hours later, the cells were fixed with methyl alcohol for 20 min, and stained with 0.5% crystal violet for 10 min. Then the cells on the upper surface of the filter were removed using a cotton swab. Five fields were imaged per Transwell insert, and the number of cells was counted using the particle counting module in Photoshop CS5 (Adobe, San Jose, CA, USA). 

### 4.13. RNA-Sequencing Analysis

For RNA-seq analysis, the exponentially grown control and B-Myb stable overexpression cells were collected, and a cDNA library was constructed and sequenced as described previously [34]. Briefly, total RNA was extracted with TRIzol reagent (Invitrogen) and treated with RNase-free DNase I (TAKARA). RNA integrity was verified by 2100 bioanalyzer (Agilent). Poly (A) mRNA was then isolated with oligo-dT beads and subjected to fragmentation. The RNA fragments were then used to synthesize double stranded cDNA using random hexamer primers followed by end-repair using T4 DNA polymerase, Klenow fragment, and T4 polynucleotide kinase, poly (A) mRNA was added using Klenow 3′ to 5′ exo-polymerase, and adapter ligation using T4 quick DNA ligase. The fragments containing adapter were then amplified by PCR and purified with magnetic beads to obtain the library. The quality and quantity of the library contents were validated by Agilent 2100 Bioanalyzer and real time PCR and then sequenced using Illumina HiSeq2000 (Illumina, San Diego, CA, USA). Short oligonucleotide alignment program (SOAP) was used to map reads for specific transcripts [35]. The expression value for each transcript was measured by reads per kilobase of transcript sequence per million mapped reads (RPKM) [36]. The transcript fold change was calculated by the formula of log^2^ (LV-control-RPKM/LV-B-Myb-RPKM). Differentially expressed genes were finally subjected to Gene Ontology Enrichment Analysis and Kyoto Encyclopedia of Genes and Genomes (KEGG) Pathway analysis [34]. 

### 4.14. Tumor Xenografts

For the tumor xenografts, 3 × 10^7^ stable control or B-Myb overexpression cells were subcutaneously injected into the dorsal flank of four to six-week old nude mice (BALB/c). Five mice were used for each group, respectively. The tumor size was measured every four days by a vernier caliper along two perpendicular axes. The volume of the tumor was calculated following the formula: volume = 1/2 × length × width^2^. Five weeks after injection, the mice were killed, and the tumor specimens were weighed. All experimental procedures involving animals were conducted based on laboratory animal protocols approved by Laboratory Animal Center of Chongqing Medical University (No. 20150727). 

### 4.15. Statistical Analysis

Data are presented as the mean ± SD of three independent experiments. All statistical analyses were conducted using the SPSS 16.0 statistical software package (SPSS Inc., Chicago, IL, USA). ANOVA, unpaired T test, and independent-samples T test were used to test for statistical significance among different groups in the in vitro experiments. Fisher's exact test was used for analyzing the immunohistochemistry (IHC) data. 

## Figures and Tables

**Table 1:** Expression of B-Myb in lung cancer determined by immunohistochemistry.

Pathological Variables	Sample No.	B-Myb IHC Staining (%)		*p* Value
		Negative	Positive	
Normal tissues	3	2 (66.7)	1 (33.3)	0.000 ***
Lung cancer tissues	69	28 (40.6)	41 (59.4)	
Pathological diagnosis				0.001 ***
Non-small cell lung cancer	62	21 (33.9)	41 (66.1)	
Small cell lung cancer	7	7 (100)	0 (0)	
Non-small cell lung cancer				0.000 ***
Adenocarcinoma	28	10 (35.7)	18(64.3)	
Atypical carcinoid	6	6 (100)	0 (0)	
Large cell carcinoma	2	1 (50)	1 (50)	
Squamous cell carcinoma	26	4 (15.4)	22 (84.6)	
Sex				0.408
Male	51	19 (37.25)	32 (62.75)	
Female	18	9 (50)	9 (50)	
Stage				1.000
I	24	10 (41.7)	14 (58.3)	
II	24	9 (37.5)	15 (62.5)	
III + IV	21	8 (38.1)	13 (61.9)	
Lymph node status				0.030 *
pN0	35	21 (60.0)	14 (40.0)	
pN1+	34	11 (32.4)	23 (67.6)	
Tumor size				0.096
T1	4	3 (75)	1 (25)	
T2	43	11 (25.6)	32 (74.4)	
T3	8	3 (37.5)	5 (62.5)	
T4	7	4 (57.1)	3 (42.9)	

Fisher’s Exact Test. * *p* < 0.05, *** *p* < 0.001. pN0, tissues with lymph node metastasis; pN1+, tissues without lymph node metastasis; IHC, immunohistochemistry.

## Supplementary Materials

Supplementary materials can be found at www.mdpi.com/1422-0067/18/4/860/s1.

## Abbreviations

NSCLC	non-small cell lung cancer
SQCC	lung squamous cell carcinoma
ADC	lung adenocarcinoma
SCLC	small cell lung cancer
Mybl2	MYB proto-oncogene like 2
COL11A1	collagen type XI alpha 1 chain
COL6A1	collagen type VI alpha 1 chain
FNT	putative formate/nitrite transporter
MMP2	matrix metallopeptidase 2
NID1	nidogen 1
FLT4	fms related tyrosine kinase 4
INSR	insulin receptor
CCNA1	cyclin A1
ERK	extracellular regulated MAP kinase
Sp1	Sp1 transcription factor
PDK1	pyruvate dehydrogenase kinase 1
