# Supplementary material for: B-Myb Is Up-Regulated and Promotes Cell Growth and Motility in Non-Small Cell Lung Cancer
Source: Int J Mol Sci. 2017 May 27;18(6):860. doi: 10.3390/ijms18060860 (PMC5485926; doi:10.3390/ijms18060860)
Supplement: Supplementary file 1 [file ijms-18-00860-s001.docx]

Supplementary Materials: B-Myb is Up-Regulated and Promotes Cell Growth and Motility in Non-Small Cell Lung Cancer

Yuelei Jin, Huifang Zhu, Wei Cai, Xiaoyan Fan, Yitao Wang, Yulong Niu, Fangzhou Song and Youquan Bu


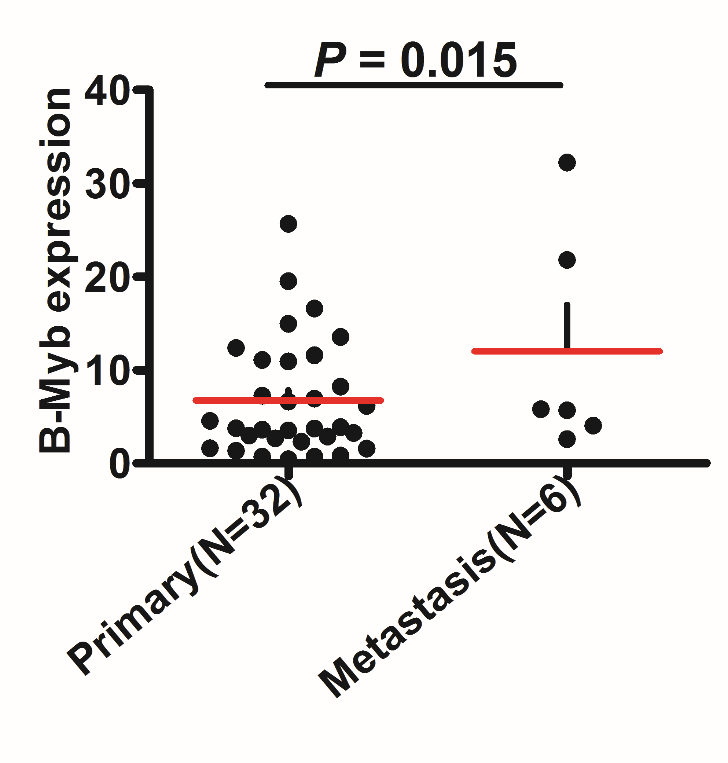


Figure S1. Expression of B-Myb was determined by qRT-PCR in samples with metastasis and with primary tumors.

**Table S1.** Gene Ontology Enrichment Analysis on genes affected by B-Myb overexpression.

| **Gene Ontology Term** | **Gene Number** | **Genes Annotated to the Term** | ***p* Value** |
| --- | --- | --- | --- |
| Extracellular matrix | 23 | *MMP11,NID1,FN1,FBLN2,GPC4,MGP,VWA1,COL11A1,FBLN1,ADAMTSL4,TNC,HMCN1,PRELP,FGF1,APLP1,ECM1,NPNT,SPARC,MMP2,COL5A1,COL6A1,PTN,LOX* | 1.10 × 10^−5^ |
| P rotein binding | 122 | *BCAM,GPX3,TRIM16,HTRA1,MGP,MFGE8,RXRA,SOCS2,FBXO2,SLIT3,PLOD1,DNAJC25,GNG10,DIP2C,SDC1,KIF26A,TNC,LPAR3,LTBP3,CTNNA2,HIST1H2AI,GTF2IP1,TUBB4A,CDKN2C,ECM1,SCNN1A,NPNT,TNNT1,DOK4,SLC22A18,COL5A1,FOSB,LIN7A,PDCD6IPP2,ARNT2,MYH14,STC1,TPPP,IGFBP3,SLC22A5,OLFML2A,CAMSAP3,GSTZ1,EPB41L1,PTPRM,LRP1,MEF2B,WBP1,DOK6,PLGLB2,ADM,FGF1,GABRB3,GATA6,MAP2,MYLK,SEPT5,GP1BB,SERPINE1,CLU,5Sep,RGS14,NRGN,ABCA1,PTN,CNTN1,CLDN1,SEMA6B,ENO2,FN1,SERPINE2,IGFBP6,SDC2,CALM3,GDF15,FOXP1,ADAMTSL4,IL22RA1,SIRPA,THBS1,TFAP4,APLP1,MYL4,ADD3,ANK1,EPHB2,GATA2,SPARC,PRKCD,JUNB,COL6A1,FCGRT,MMP2,IRF9,ABCD1,BNIP3,LIMCH1,MLXIPL,IGFBP2,ABLIM3,NPTXR,CLIP2,SPAG4,PFKFB4,DNM1,EPOR,GPC4,TMX2,CTNND1,SMARCD3,ADRB2,TAF4B,FOS,DKK1,POP1,INSR,MYBBP1A,PAK3,PALM,PLGLB1,GNAO1,MPP2,TMEM173,CORO2B* | 4.86 × 10^−5^ |
| Insulin-like growth factor binding | 4 | *IGFBP2,INSR,IGFBP6,IGFBP3* | 0.00032 |
| Growth factor binding | 9 | *INSR,TRIM16,IGFBP6,HTRA1,COL6A1,COL5A1,IGFBP2,THBS1,IGFBP3* | 0.00696 |
| Response to steroid hormone stimulus | 20 | *GPX3,ENO2,MFGE8,RXRA,SOCS2,SLIT3,SDC1,FOS,ADM,THBS1,TFAP4,INSR,GATA6,JUNB,FOSB,MMP2,IGFBP2,SERPINE1,ARNT2,STC1* | 0.00061 |
| Developmental process | 99 | *PCDHAC2,MGP,MFGE8,RXRA,SOCS2,SLIT3,PLOD1,COL11A1,CRELD1,S1PR1,SDC1,KIF26A,TNC,LTBP3,CTNNA2,GTF2IP1,CDKN2C,SPEG,ECM1,EGFL8,NPNT,DOK4,COL5A1,RAB26,NOTCH3,ARNT2,CPE,RPL13AP20,STC1,LOX,CELSR2,PTPRM,LRP1,MEF2B,IGSF8,PLGLB2,ADM,PRELP,FGF1,GATA6,MAP2,MYLK,SERPINE1,PLXND1,H3F3A,NRGN,ABCA1,PTN,SEMA6B,SERPINE2,FN1,SDC2,CALM3,ETNK2,SEMA3E,RABP2,FOXP1,SELENOP,BZW2,THBS1,TNS3,MYL4,APLP1,ANK1,EPHB2,OBSL1,GATA2,MYC,PRKCD,JUNB,MMP2,IGFBP2,MLXIPL,SALL3,CTSH,TLE2,DPYSL5,EPOR,GPC4,TMX2,CTNND1,SMARCD3,EPHA5,TCF7,ADRB2,FOS,PHYH,NR2F1,DKK1,INSR,PCDHB11,PLGLB1,GNAO1,SLC7A5,SEMA4G,NFATC4,MYRF,SLC30A1,TRO* | 0.01657 |

**Table S2.** List of primer sequences used for qRT-PCR analysis.

| **Gene Name** | **Primer Sequences** |
| --- | --- |
|  |  |
| *GAPDH* | GAPDH F (833): ACCTGACCTGCCGTCTAGAA  GAPDH R (1060): TCCACCACCCTGTTGCTGTA |
| *B-Myb* | B-Myb F (1067): AGAAACGAGCCTGCCTGCCTTACA  B-Myb R (1220): AGATGGTTCCTCAGGGAGGT |
| *FLT4* | FLT4 F (2870): AAGTACGGCAACCTCTCCAA  FLT4 R (3086): CCTCAGCTTCTTGGTCTGGA |
| *FN1* | FN1 F (2294): GTATACGAGGGCCAGCTCAT |
|  | FN1 R (2489): CCCAGGAGACCACAAAGCTA |
| *MMP2* | MMP2 F (1073): TATGACAGCTGCACCACTGA |
|  | MMP2 R (1317): TCATCGTAGTTGGCTGTGGT |
| *NID1* | NID1 F (3440): GACTGACCTTCGATGCGTTC |
|  | NID1 R (3637): CAAGATCGAGAGCAACCACG |
| *INSR* | INSR F (3372): TCTCAGTGCCAGTGATGTGT |
|  | INSR R (3525): TGCCTCACCCTTGATGATGT |
| *CCNA1* | CCNA1 F (493): ACCCCAAGAGTGGAGTTGTG |
|  | CCNA1 R (690): GGAAGGCATTTTCTGATCCA |
| *COL11A1* | COL11A1 F (1275): GCCTGGTATGCTTGTCGAAG  COL11A1 R (1508): CCTGAGCAGAGATGGTTGG |
| *COL6A1* | COL6A1 F (2164): AAGGAAGCCATCAAGAGCCT  COL6A1 R(2381): AACACGTCTTTGATGCCCAC |

**Table S3.** Lung cancer tissue microarray information.

| **#** | **Pos** | **Sx** | **Age** | **Pathological Diagnosis** | **Grade** | | **Stage** | **TNM** | **Scores** | |
| --- | --- | --- | --- | --- | --- | --- | --- | --- | --- | --- |
| 1 | 1A | F | 58 | Adenocarcinoma | | 1 | IIIa | T3N1M0 | | 2 |
| 2 | 1B | M | 59 | Adenocarcinoma | | 1 | II | T2N1M0 | | 2 |
| 3 | 1C | M | 44 | Adenocarcinoma | | 2 | I | T1N0M0 | | 0 |
| 4 | 1D | F | 62 | Adenocarcinoma | | 2 | II | T2N1M0 | | 0 |
| 5 | 1E | M | 60 | Adenocarcinoma | | 2 | IV | T2N1M1 | | 2 |
| 6 | 1F | F | 61 | Adenocarcinoma | | 1 | II | T2N1M0 | | 3 |
| 7 | 1G | M | 57 | Adenocarcinoma | | 2 | IIIa | T2N2M0 | | 3 |
| 8 | 1H | M | 57 | Adenocarcinoma | | – | I | T2N0M0 | | 3 |
| 9 | 1I | F | 52 | Adenocarcinoma | | 2 | II | T2N1M0 | | 3 |
| 10 | 2A | F | 50 | Adenocarcinoma | | 3 | II | T2N1M0 | | 0 |
| 11 | 2B | M | 72 | Adenocarcinoma | | 3 | I | T2N0M0 | | 3 |
| 12 | 2C | M | 69 | Adenocarcinoma | | 3 | I | T2N0M0 | | 3 |
| 13 | 2D | M | 56 | Adenocarcinoma | | 3 | IIIa | T3N1M0 | | 5 |
| 14 | 2E | F | 70 | Adenocarcinoma | | 3 | I | T2N0M0 | | 2 |
| 15 | 2F | M | 63 | Adenocarcinoma | | 3 | IIIa | T3N0M0 | | 0 |
| 16 | 2G | M | 51 | Adenocarcinoma | | 3 | II | T2N1M0 | | 3 |
| 17 | 2H | F | 38 | Adenocarcinoma | | 3 | IIIa | T2N2M0 | | 2 |
| 18 | 2I | M | 71 | Adenocarcinoma | | 3 | I | T2N0M0 | | 0 |
| 19 | 3A | F | 48 | Adenocarcinoma | | 3 | I | T2N0M0 | | 0 |
| 20 | 3B | F | 56 | Adenocarcinoma | | 3 | IIIa | T3N0M0 | | 0 |
| 21 | 3C | F | 57 | Adenocarcinoma | | 2–3 | IIIb | T2N3M0 | | 3 |
| 22 | 3D | M | 66 | Atypical carcinoid | | – | I | T2N0M0 | | 0 |
| 23 | 3E | M | 75 | Atypical carcinoid | | – | I | T2N0M0 | | 0 |
| 24 | 3F | F | 36 | Atypical carcinoid | | – | I | T2N0M0 | | 0 |
| 25 | 3G | M | 52 | Atypical carcinoid | | – | IIIb | T4N2M0 | | 0 |
| 26 | 3H | M | 61 | Atypical carcinoid | | – | I | T1N0M0 | | 0 |
| 27 | 3I | M | 70 | Atypical carcinoid | | – | IIIb | T4N0M0 | | 0 |
| 28 | 4A | F | 67 | Adenocarcinoma | | 2 | IIIa | T3N1M0 | | 5 |
| 29 | 4B | M | 40 | Large cell carcinoma | | – | IIIb | T4N0M0 | | 3 |
| 30 | 4C | M | 64 | Large cell carcinoma | | – | IIIb | T4N0M0 | | 0 |
| 31 | 4D | M | 70 | Papillary adenocarcinoma | | 1 | I | T1N0M0 | | 0 |
| 32 | 4E | M | 46 | Papillary adenocarcinoma | | 1 | IIIb | T4N0M0 | | 0 |
| 33 | 4F | M | 46 | Papillary adenocarcinoma | | 1 | I | T2N0M0 | | 2 |
| 34 | 4G | M | 61 | Papillary adenocarcinoma | | 2 | I | T2N0M0 | | 2 |
| 35 | 4H | F | 55 | Papillary adenocarcinoma | | 2 | II | T2N1M0 | | 0 |
| 36 | 4I | M | 40 | Papillary adenocarcinoma | | 2 | I | T2N0M0 | | 5 |
| 37 | 5A | M | 62 | Small cell carcinoma | | – | I | T2N0M0 | | 0 |
| 38 | 5B | F | 63 | Small cell carcinoma | | – | II | T2N1M0 | | 0 |
| 39 | 5C | M | 57 | Small cell carcinoma | | – | II | T2N1M0 | | 0 |
| 40 | 5D | M | 60 | Small cell carcinoma | | – | II | T2N1M0 | | 0 |
| 41 | 5E | F | 54 | Small cell carcinoma | | – | I | T1N0M0 | | 0 |
| 42 | 5F | M | 56 | Small cell carcinoma | | – | II | T2N1M0 | | 0 |
| 43 | 5G | M | 50 | Small cell carcinoma | | – | IIIa | T3N2M0 | | 0 |
| 44 | 5H | M | 68 | Squamous cell carcinoma | | – | I | T2N0M0 | | 3 |
| 45 | 5I | M | 59 | Squamous cell carcinoma | | 1 | II | T2N1M0 | | 5 |
| 46 | 6A | M | 54 | Squamous cell carcinoma | | 1 | I | T2N0M0 | | 3 |
| 47 | 6B | M | 71 | Squamous cell carcinoma | | 2 | II | T2N1M0 | | 5 |
| 48 | 6C | M | 70 | Squamous cell carcinoma | | 2 | I | T2N0M0 | | 5 |
| 49 | 6D | M | 57 | Squamous cell carcinoma | | 2 | I | T2N0M0 | | 3 |
| 50 | 6E | M | 68 | Squamous cell carcinoma | | 2 | II | T2N1M0 | | 2 |
| 51 | 6F | M | 50 | Squamous cell carcinoma | | 2 | II | T2N1M0 | | 5 |
| 52 | 6G | M | 67 | Squamous cell carcinoma | | 2 | II | T2N1M0 | | 5 |
| 53 | 6H | M | 68 | Squamous cell carcinoma | | 2 | I | T2N0M0 | | 5 |
| 54 | 6I | M | 53 | Squamous cell carcinoma | | 2 | II | T2N1M0 | | 0 |
| 55 | 7A | M | 48 | Squamous cell carcinoma | | 2 | IIIa | T2N2M0 | | 3 |
| 56 | 7B | M | 61 | Squamous cell carcinoma | | 2 | I | T2N0M0 | | 3 |
| 57 | 7C | M | 71 | Squamous cell carcinoma | | 2 | IIIb | T4N0M0 | | 3 |
| 58 | 7D | M | 75 | Squamous cell carcinoma | | 2 | II | T2N0M0 | | 3 |
| 59 | 7E | M | 47 | Squamous cell carcinoma | | 3 | II | T2N1M0 | | 0 |
| 60 | 7F | M | 58 | Squamous cell carcinoma | | 3 | II | T2N1M0 | | 5 |
| 61 | 7G | M | 74 | Squamous cell carcinoma | | 3 | IIIa | T3N2M0 | | 5 |
| 62 | 7H | M | 62 | Squamous cell carcinoma | | 3 | II | T2N0M0 | | 3 |
| 63 | 7I | M | 66 | Squamous cell carcinoma | | 3 | II | T2N1M0 | | 5 |
| 64 | 8A | M | 64 | Squamous cell carcinoma | | 3 | IIIa | T3N1M0 | | 5 |
| 65 | 8B | M | 59 | Squamous cell carcinoma | | 3 | II | T2N0M0 | | 4 |
| 66 | 8C | F | 38 | Squamous cell carcinoma | | 3 | IIIb | T4N1M0 | | 3 |
| 67 | 8D | M | 70 | Squamous cell carcinoma | | 3 | IIIa | T3N0M0 | | 0 |
| 68 | 8E | F | 53 | Squamous cell carcinoma | | 3 | I | T1N0M0 | | 5 |
| 69 | 8F | F | 57 | Squamous cell carcinoma | | 3 | II | T2N1M0 | | 0 |
| 70 | 8G | M | 26 | Normal lung tissue | | – | – | – | | 0 |
| 71 | 8H | M | 51 | Normal lung tissue | | – | – | – | | 3 |
| 72 | 8I | M | 52 | Normal lung tissue | – | | – | – | | 0 |
| 73 | 8J | - | - | Array marker | – | | – | – | | 5 |

TNM: tumor-node-metastasis; Pos: position.

**Table S4.** Antibodies used in the present study.

| **Protein Name** | **Manufacture (cat. number)** | | **Applications (working dilution)** | **Website Link** |
| --- | --- | --- | --- | --- |
| GAPDH | Xianzhi Bio  (AB-P-R 001) | IB (1:5000) | | http://www.goodhere.com/showproduct.asp?id=320&classid=34&nid=2 |
| B-Myb | Santa Cruz  (N-19):sc-724 | IB (1:500) | | <http://www.scbt.com/datasheet-724-b-myb-n-19-antibody.html> |
| B-Myb | Santa Cruz  (H-115):sc-13028 | IHC (1:50) | | http://www.scbt.com/datasheet-13028-b-myb-h-115-antibody.html |
| ERK | CST(4695S) | IB (1:2000) | | http://www.cst-c.com.cn/products/4695.html |
| p-ERK | CST(4370S) | IB (1:2000) | | http://www.cst-c.com.cn/products/4370.html |
| p-Akt | Ruiying Bio  (RLP0006) | IB (1:500) | | http://rlgene.com/showproduct.asp?/1_439 |
| antiMouse secondary antibody | Abgent  (ASS1007) | IB (1:5000) | | http://www.abgent.com/products/ASS1007-Goat-Anti-Mouse-IgGHL-Human-ads-HRP-Secondary-Antibody |
| antiRabbit secondary antibody | Abgent  (ASS1009) | IB (1:5000) | | http://www.abgent.com/products/ASS1009-Goat-Anti-Rabbit-IgGHL-MouseHuman-ads-HRP-Secondary-Antibody |

IB: immunoblot; IHC: immunohistochemistry; GAPDH: glyceraldehyde-3-phosphate dehydrogenase.
